# Cut-Off Value of Serum Uric Acid for Development of Gout Disease in Patients with Multiple Co-Morbidities

**DOI:** 10.31138/mjr.32.3.243

**Published:** 2021-09-30

**Authors:** Firdevs Ulutaş, Aslı Bozdemir, Nevzat Atalay Çelikyürek, Canan Albayrak Yaşar, Veli Çobankara

**Affiliations:** 1 Division of Rheumatology, Department of Internal Medicine, Pamukkale University Faculty of Medicine, Denizli, Turkey; 2 Department of Internal Medicine, Pamukkale University Faculty of Medicine, Denizli, Turkey; 3 Department of Public Health, Pamukkale University Faculty of Medicine, Denizli, Turkey

**Keywords:** hyperuricemia, gout, co-morbid disease, cut-off

## Abstract

**Objective:**

This study aimed to determine the association between hyperuricemia, comorbid diseases and risk of developing gout disease in a Turkish population in a long follow-up period.

**Methods:**

A total of 2000 Turkish adults were cross-sectionally analysed for serum urate levels in 2009 at the Pamukkale University Faculty of Medicine. All patients on regular follow-up at our tertiary health center (n=1322) were included in this study. Demographic features (age and gender), comorbid clinical conditions, and medication use were noted. The risk of developing gout and the determinant factors were investigated. Multivariate logistic regression analysis was performed to analyse causative effects of factors while receiver operating characteristic (ROC) curve analysis was used to identify optimal cut-off values of uric acid level for predicting the development of gout.

**Results:**

Among 1322 patients, the mean age was 56.9 (SD:14.68) years. 57.3% (n=758) of the patients were female. The most common co-morbid disease was chronic kidney disease (581, 43%), followed by hypertension (522, 39.4%). Gout developed in 25 patients (1.8%). Gout development risk and presence of all the above comorbidities were significantly higher in patients with serum urate ≥7mg/dl compared with <7mg/dl. Male gender and not using urate lowering drugs were the main risk factors for developing gout disease. ROC analysis of serum uric acid levels identified a cut-off value of >7 (AUC: 0.776, sensitivity 86.96%, specificity 66.74%).

**Conclusion:**

There is still a dilemma concerning the culprit effects of both comorbidities and hyperuricemia on the risk of developing gout disease.

## INTRODUCTION

Gout is an increasingly common chronic inflammatory disease that manifests as flares of acute inflammatory arthritis and/or clinical manifestations of chronic urate crystal deposition.^1^ A population-based epidemiological study revealed the estimated prevalence of gout as 0.31% for subjects ≥ 20 years of age, and 0.72% for subjects ≥ 40 years of age in an urban area of Turkey.^2^ The burden of gout has increased in the last decades in industrialised countries. This increased incidence is attributed to changes in lifestyle, to related comorbidities including obesity and metabolic syndrome and to prolonged lifespan.^3^

The normal reference range of serum uric acid is determined by evaluating its variable levels among healthy subjects who have no clinical evidence of hyperuricemia. The cut-off value of hyperuricemia varies due to sex-specific effects (sex–diet and sex–gene interactions) in women and men.^4^ Although there is no well-defined accepted definition of hyperuricemia today, exact evidence of a relationship between degree of hyperuricemia and risk of developing gout has been reported.^5^ Campion EW et al. investigated the relationship between serum uric acid and the development of gout in 2046 initially healthy men for 15 years. They found a positive correlation and that the cumulative incidence of gout with serum urate levels of 9 mg/dL or greater was 22 percent after five years.^6^ Animal models and cell culture studies have shown that hyperuricemia also plays a main culprit role in the underlying pathophysiological mechanism in many “cardio-nephro-metabolic” disorders without the deposition of monosodium urate crystals.^7^ For example, increased serum uric acid leads to high blood pressure, initially via direct action on smooth muscle and vascular endothelial cells, then plays a role as an independent risk factor for poor cardiovascular prognosis.^8^

Many observational studies have also noted that hyperuricemia alone is not a sufficient risk factor for development of gout. These observational studies showed 4–5 co-morbidity clusters in gout patients.^9^ In addition, Mendelian randomisation studies showed new genome-wide significant loci associated with serum uric acid concentration.^10^ Several common comorbidities also have a higher prevalence among patients with asymptomatic hyperuricemia. Therefore, the causal relationships between gout, relevant comorbidities and hyperuricemia are quite complex and their interrelationships are not yet fully understood due to heteregeneous study design and varied populations. There has been no observational study that investigated the association between hyperuricemia, relevant comorbidities and development of gout with long term follow-up in the Turkish population. We aimed to determine the risk cut-off value of serum uric acid for development of gout in patients with multiple comorbidities.

## MATERIALS AND METHODS

This observational cohort study was designed to quantify the risk of developing gout disease in patients with hyperuricemia and multiple co-morbidities among 2000 Turkish adults whose serum uric acid levels were cross-sectionally analysed in 2009. All of the patients (n=1322) enrolled in this study were on regular follow-up for eleven years in our tertiary health center.

Inclusion criteria were defined as: having regular follow-up for the time period studied. Exclusion criteria were defined as: having no sufficient medical data due to irregular follow-up, death of the patient in the follow-up period, or having gout diagnosed initially.

835 patients with baseline levels of SUA<7.0 mg/dl and 487 patients with SUA≥7 mg/dl were included. We called all of the subjects by telephone and questioned them on complaints of arthralgia in any joint, morning stiffness, or tophi. All of the suspected individuals were invited to the hospital for physical examination and repeated laboratory measurements including serum uric acid and acute phase reactants. Gout was clinically diagnosed by the same rheumatologist based on history and physical examination according to the current clinical classification criteria.^11^ For patients who were diagnosed with gout arthritis, family history, alcohol consumption, diuretic use, urate lowering drug use and obesity were carefully investigated.

Demographic features (age and gender), comorbid clinical conditions including hypertension, diabetes mellitus, hyperlipidemia, chronic kidney disease, nephrolithiasis and cardiovascular disease, and medication use were noted exactly. Mean systolic blood pressure ≥140 mmHg or mean diastolic blood pressure ≥90 mmHg, fasting serum glucose levels ≥126 mg/dl, total cholesterol ≥200 mg/dl or low-density cholesterol (LDL-C) ≥130 mg/dl were accepted as positive cut-off values for hypertension, diabetes mellitus, and hyperlipidemia, respectively, whereas history of coronary artery disease, heart failure, and/or using relevant drugs defined cardiovascular disease. Having treatment for cardiovascular disease was also accepted as having this co-morbidity. Individuals who had an estimated-glomerular filtration rate (e-GFR) below 60 mL/min per 1.73 m^2^ were accepted as having chronic kidney disease. For defining nephrolithiasis, presence of positive imaging was accepted.

The study protocol conformed to the ethical guidelines of the 1975 Declaration of Helsinki as reflected in a prior approval by the local Ethics Committee of our health center. Informed consent forms were obtained from patients in this study.

### Statistical Analysis

For statistical analysis, SPSS 17.0 (SPSS Inc. Released 2008. SPSS Statistics for Windows, Version 17.0. Chicago: SPSS Inc.) was used. Descriptive statistics are given as mean ± standard deviation for continuous variables. Numbers and percentages are used for categorical variables. Normality of numerical variables was checked with Kolmogorov-Smirnov and Shapiro-Wilk tests. Mann Whitney U test was used for comparison of two independent groups and Kruskal-Wallis test was used for comparison of three or more independent groups. For comparison of differences between categorical variables, Chi-Square test was used. Multivariate logistic regression analyses were performed to analyse factors that had an impact on the development of gout. Variables were adjusted for age and sex. Receiver operating characteristic (ROC) curve analysis was used to determine optimal cut-off values of uric acid level for predicting the development of gout. Area under curve (AUC) was calculated using the DeLong method (MedCalc Statistical Software Trial version-MedCalc Software bvba, Ostend, Belgium; http://www.medcalc.org; 2015) with Youden’s index and a confidence interval of 95%. p<.05 (2-sided) was considered statistically significant in all statistical analyses.

## RESULTS

A total of 1322 patients were enrolled in this study. The mean age was 56.9 years (SD:14.68) in the whole study group. 57.3% (n=758) of patients were female. The most common comorbid disease was chronic kidney disease (CKD) (n=581, 43%), followed by hypertension (n=522, 39.4%), hyperlipidemia (n=488, 36.8%), diabetes mellitus (n=467, 35.3%), cardiovascular disease (n=238, 18%), and nephrolithiasis (n=44, 3.3%). 7.6% of patients (n=101) were initially on urate lowering therapy (**Table 1**). Gout developed in 25 patients (1.8%). 14 (56%) patients diagnosed with gout initially presented with podagra joint, and a family history of gout was present for 7 (28%) patients (**Table 1**).

**Table 1.** Patient characteristics in the whole study group (n=1322).

**Patient characteristics**	**n (%)**
**Serum uric acid (mg/dl)**	
7mg/dl ≥	487 (36.8)
7mg/dl <	835 (63.2)
**Levels of uric acid (mg/dl)**	
7mg/dl <	835 (63.2)
7mg/dl −7.9 mg/dl	270 (20.4)
8mg/dl −8.9 mg/dl	146 (11)
9mg/dl ≥	71 (5.4)
**Age**	Mean: 56.93 (SD: 14.68)
**Gender**	
Male	564 (42.7)
Female	758 (57.3)
**Chronic kidney disease**	581 (43.9)
**Diabetes mellitus**	467 (35.3)
**Hyperlipidaemia**	488 (36.8)
**Hypertension**	522 (39.4)
**Nephrolithiasis**	44 (3.3)
**Cardiovascular disease**	238 (18)
**Alcohol use**	14 (1.1)
**Urate lowering drugs (Allopurinol:100, Febuxostat:1)**	101 (7.6)
**Gout development in whole group**	25 (1.8)
**Podagra presentation in gout**	14 (56)
**Family history in gout**	7 (28)

**Figure 1.**
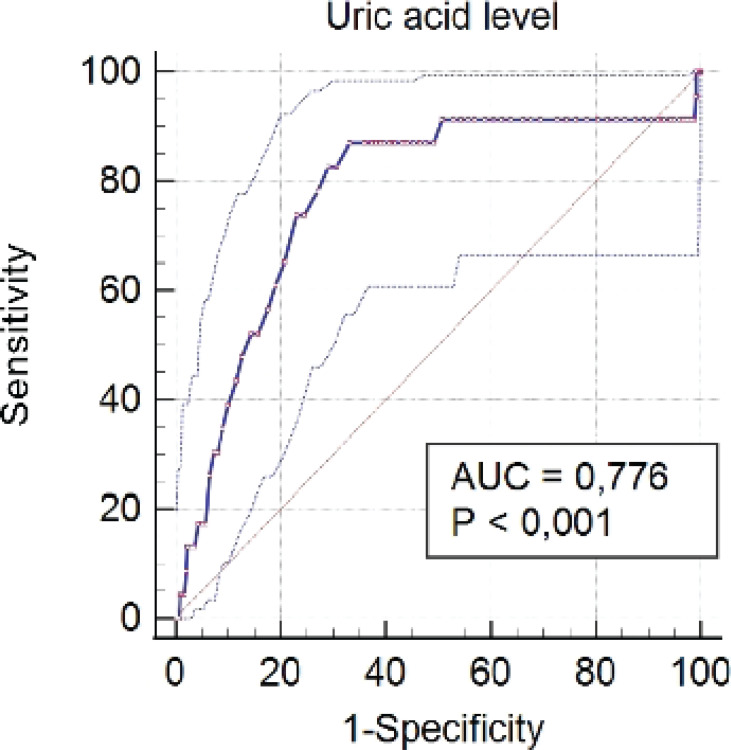
ROC analysis of blood uric acid levels for the development of gout (The cut-off value of uric acid was defined as >7 sensitivity 86.96%, specificity 66.74%, [0.752 – 0.798,95% CI]) ROC: receiver operating curve; AUC: area under curve; CI: confidence interval.

Gout development risk, male dominance, presence of all the above comorbidities, and using ULDs were significantly higher in patients with serum urate ≥7mg/dl compared with <7mg/dl (**Table 2**). The possibility of diagnosis with CKD, NL and CVD was significantly higher in patients with SUA ≥9 mg/dl compared with lower serum urate levels (**Table 3**). Male gender and not using urate lowering drugs were the main risk factors for developing gout disease (**Table 4**). There was no statistically significant difference between patients with and without gout based on the presence of concurrent comorbidities, except male predominance for gout patients. ROC analysis of serum uric acid levels revealed a cut-off value of >7 (AUC: 0.776, sensitivity 86.96%, specificity 66.74%) (**Table 5**, **Figure 1**).

**Table 2.** Comparison of patient characteristics between groups determined by serum uric acid levels (UA ≥7mg/dl or UA <7mg/dl).

	**UA ≥7mg/dl n (%)**	**UA <7mg/dln (%)**	**p value**
Gender			**<0,001**
Male	325 (66.7)	239 (28.6)	
Female	162 (33.3)	596 (71.4)	
CKD	342 (70.2)	239 (28.6)	**<0.001**
DM	249 (51.1)	218 (26.1)	**<0.001**
HL	211 (43.3)	277 (33.2)	**<0.001**
HT	303 (62.2)	219 (26.2)	**<0.001**
NL	29 (6)	15 (1.8)	**<0.001**
CVD	132 (27.1)	106 (12.7)	**<0.001**
Alcohol	9 (1.8)	5 (0.6)	0.063
Gout development	21 (4.3)	4 (0.5)	**<0.001**
ULDs			**<0.001**
Allopurinol	89 (18.3)	11 (1.3)	
Febuxostat	1 (0.2)	0 (0)	

UA: uric acid; CKD: chronic kidney disease; DM: diabetes mellitus, HL: hyperlipidaemia; HT: hypertension; NL: nephrolithiasis; CVD: cardiovascular disease, ULDs: urate lowering drugs.

**Table 3.** Comparison of patients with serum uric acid levels above UA ≥7mg/dl in three subgroups.

		**UA ≥ 7mg/dl – 7.9 mg/dl (n, %)**	**UA ≥ 8 mg/dl – 8.9 mg/dl (n, %)**	**UA ≥ 9 mg/dl (n, %)**	**P value**
Gender	Male	178 (65.9)	99 (67.8)	48 (67.6)	0,914
	Female	92 (34.1)	47 (32.2)	23 (32.4)	
CKD		174 (64.4)	**114 (78.1)**	**54 (76.1)**	**0.008**
DM		147 (54.4)	69 (47.3)	33 (46.5)	0.262
HL		112 (41.5)	61 (41.8)	38 (53.5)	0.172
HT		169 (62.6)	86 (58.9)	48 (67.6)	0.455
NL		15 (5.6)	4 (2.7)	**10 (14.1)**	**0.004**
CVD		63 (23.3)	**44 (30.1)**	**25 (35.2)**	**0.083**
Alcohol		4 (1.5)	3 (2.1)	2 (2.8)	0.740
Gout		9 (3.3)	8 (5.5)	4 (5.6)	0.494
ULDs	Alloprinol	40 (14.8)	22 (15.1)	**27 (38)**	**<0.001**
	Febuxostate	0 (0)	0 (0)	1 (1.4)	

UA: uric acid; CKD: chronic kidney disease; DM: diabetes mellitus; HL: hyperlipidaemia; HT: hypertension; NL: nephrolithiasis; CVD: cardiovascular disease.

**Table 4.** Odds ratio between variables.

	**Adjusted OR**	**95% CI**		**p value**
**Age**	1.027	0.997	1.058	0.074
**Male Gender**	6.363	2.149	18.840	0.001
**CKD**	0.704	0.299	1.658	0.422
**DM**	1.448	0.620	3.383	0.392
**HL**	1.357	0.587	3.137	0.475
**HT**	0.497	0.198	1.248	0.136
**NL**	2.194	0.482	9.993	0.310
**CVD**	1.313	0.503	3.427	0.578
**Not using ULDs**	29.772	11.238	78.870	<0.001

Odds ratio was adjusted for age and sex.

UA: uric acid; CKD: chronic kidney disease; DM: diabetes mellitus; HL: hyperlipidaemia; HT: hypertension; NL: nephrolithiasis; CVD: cardiovascular disease; ULDs: urate lowering drugs.

**Table 5.** Presence of variables in gout and non-gout patients.

**Gout (n, %) (n=25)**		**Presence of gout disease**		**P value**
		**Non-gout (n, %) (n=1297)**		
Gender	Male	20 (80)	544 (41)	<0.001
	Female	5 (20)	753 (58)	
**CKD**		10 (40)	571 (44)	1.000
**DM**		12 (48)	455 (35)	0.137
**HL**		11 (44)	477 (36.7)	0.381
**HT**		8 (32)	514 (39.6)	0.802
**NL**		4 (16)	45 (3.4)	0.177
**CVD**		7 (28)	231 (17.8)	0.163
**Alcohol**		0 (0)	14 (1.1)	1.000

CKD: chronic kidney disease; DM: diabetes mellitus; HL: hyperlipidaemia; HT: hypertension; NL: nephrolithiasis; CVD: cardiovascular disease.

## DISCUSSION

We found that all of the comorbidities including CKD, HT, HL, NL, DM, CVD, and risk of developing gout were more commonly seen in patients having SUA ≥ 7 mg/dl compared with < 7 mg/dl. The risk cut-off value for development of gout was defined as >7 mg/dl for patients with multiple co-morbidities. The presence of CKD, NL or CVD had a high prevelance among patients with SUA ≥ 9 mg/dl, even if they were more commonly treated with ULDs. Firstly, we noticed again that hyperuricemia was also an important etiological culprit factor for common morbidities such as cardiovascular disease, chronic kidney disease, and NL, similar to recent experimental and epidemiologic studies.^12^ Secondly, this result reinforced our opinion of the real beneficial effects of ULDs on insulin resistance, hypertension, and development of gout since a few studies have shown that hyperuricemic control with the available drugs may ameliorate some of these related conditions.^13^ Allopurinol-treated individuals with asymptomatic hyperuricemia showed improvement in insulin resistance, fasting blood glucose, HOMA-IR index and serum high-sensitivity C-reactive protein.^14^ It was also shown that allopurinol alleviates hypertension and proteinuria in patients with metabolic syndrome.^15^ In addition, ULDs prevent attacks in high risk patients who suffer from gout**.**

**Öztürk** MA et al. showed that the most common clinical comorbidity was hypertension (in 53.5% of patients) in patients with gout in Turkey.^16^ In a population cohort, hypertension, chronic kidney disease, obesity, diabetes mellitus, nephrolithiasis, and heart failure were substantially higher in patients with gout than without gout, and the overall comorbidity prevalence was highest when gout and hyperuricemia were present together.^17^ There was no statistical significance for the presence of comorbidities in gout and non-gout patients but the likelihood of having DM, CKD, or HL was higher in gout patients. This can be explained by the very limited number of gout patients in the whole cohort.

Data from the Unites States National Health and Nutrition Examination Survey (NHANES) 2007–2008 study showed a gout prevalence of 3.9% (5.9% for men; 2.0% for women), but a higher hyperuricemia prevalence of 21.4% (21.2% for men; 21.6% for women).^17^ The prevalence of hyperuricemia has also risen steadily over the last decades, as has gout.^18^ Ultimately, gout developed in a minority of patients with hyperuricemia, as shown in our study. Other reasons for this result may be explained by female dominance in the whole cohort and being on treatment with ULDs initially. Today most of the gene studies have been focused on genetic markers of susceptibility to gout related to renal excretion of uric acid.^19^ Genome-wide association studies (GWAS) have also identified the role of the kidneys, gut and liver as sites of urate regulation. In addition, genetic variants that predict responsiveness to therapies are still being researched. As a result, comorbidities and hyperuricemia have been identified as additional culprit factors for the development of gout in patients with genetic susceptibility.^20^

Currently, asymptomatic hyperuricemia alone is not an indication for urate lowering therapy. There have not been enough randomised placebo-controlled trials that assessed the benefits of treating in the asymptomatic phase. Masayuki Hakoda et al have noticed that the prevalence of male patients with asymptomatic hyper-uricemia under ULDs was significantly increased (2.14%) in recent years (during 2010–2014), and was higher than that of gout in Japan.^21^ In our study, the presence of gout disease was similar in the three subgroups among patients with serum urate ≥ 7 mg/dl. This condition may be explained by the higher rate of patients on ULDs in the subgroup with serum urate ≥ 9 mg/dl.

A few limitations were present in this study. First, this cross-sectional observational study could not reveal a clear causal relationship. Patients on initial ULDs treatment were included, which led to heterogeneity of patients in the groups.

## CONCLUSION

Although the cut-off value of serum uric acid was >7 for risk of gout development in patients with multiple comorbidities, there is still a dilemma as to which serum uric acid level plays a significant role in developing gout. Also, culprit effects of each comorbidity are not well defined due to cluster effects. We hope future studies, especially related to genome-wide associations and culprit factors for gout disease, will enable us to be better informed.
